# Effects of lead stress on the growth, physiology, and cellular structure of privet seedlings

**DOI:** 10.1371/journal.pone.0191139

**Published:** 2018-03-01

**Authors:** Jian Zhou, Zhaopei Zhang, Yichuan Zhang, Yuan Wei, Zeping Jiang

**Affiliations:** 1 School of Horticulture and Landscape Architecture, Henan Institute of Science and Technology, Xinxiang Henan, China; 2 Research Institute of Forestry Chinese Academy of Forestry, Beijing, China; 3 Experimental Center of Henan Institute of Science and Technology, Xinxiang Henan, China; Hainan University, CHINA

## Abstract

In this study, we investigated the effects of different lead (Pb) concentrations (0, 200, 600, 1000, 1400 mg kg^-1^ soil) on the growth, ion enrichment in the tissues, photosynthetic and physiological characteristics, and cellular structures of privet seedlings. We observed that with the increase in the concentrations of Pb, the growth of privet seedlings was restricted, and the level of Pb ion increased in the roots, stem, and leaves of the seedlings; however, most of the ions were concentrated in the roots. Moreover, a decreasing trend was observed for chlorophyll a, chlorophyll b, total chlorophyll, net photosynthesis (Pn), transpiration rate (Tr), stomatal conductance (Gs), sub-stomatal CO_2_ concentration (Ci), maximal photochemical efficiency (Fv/Fm), photochemical quenching (qP), and quantum efficiency of photosystem II (ΦPSII). In contrast, the carotene levels, minimum fluorescence (F_0_), and non-photochemical quenching (qN) showed an increasing trend. Under Pb stress, the chloroplasts were swollen and deformed, and the thylakoid lamellae were gradually expanded, resulting in separation from the cell wall and eventual shrinkage of the nucleus. Using multiple linear regression analysis, we found that the content of Pb in the leaves exerted the maximum effect on the seedling growth. We observed that the decrease in photosynthetic activation energy, increase in pressure because of the excess activation energy, and decrease in the transpiration rate could result in maximum effect on the photosynthetic abilities of the seedlings under Pb stress. Our results should help in better understanding of the effects of heavy metals on plants and in assessing their potential for use in bioremediation.

## Introduction

With rapid global economic growth, pollutants containing heavy metals can enter soil through various routes. Lead (Pb) is the most common heavy metal pollutant in soil, affecting vast land area [1]. Lead and its compounds remain stable for long periods of time in soil, are difficult to dissociate, and eventually accumulate in the human body through the food chain [2], thereby, threatening the human health. For instance, when Pb concentrations in blood exceed 40 μg dL^-1^ in infants, hemoglobin synthesis is blocked, which results in severe anemia [3]. To mitigate the deleterious effects of Pb pollution in soil, chemical, biological, botanical, and some other methods are used. Among these, the methods that make use of plants have received extensive attention because they are low-cost, environment friendly, and effective [4]. Many research groups have undertaken large-scale research projects for understanding the mechanisms involved in bioremediation and to evaluate its potential applications [5–11].

Most of the Pb ions absorbed by plants from the soil remain concentrated in the roots, and a small portion is transferred to the stems and leaves [10]. In the plant tissues, active Pb ions exert toxic effects and directly damage the photosynthetic systems [12, 13]; consequently, the developmental processes of the plants, such as growth, mineral absorption, and seed germination, are affected [5, 13–15]. Santos et al. [16] investigated the response of *Hypnea musciformis* to Pb stress, and reported that the photosynthetic ability, measured in terms of the maximum photosynthetic efficiency of photosystem II (Fv/Fm) and electron transport rate (ETR), of plants under stress was relatively stable, and was not significantly different from that of the plants grown under normal conditions. They speculated that this could be because of an increase in starch synthesis in chloroplasts, which might serve as a stored food, ensuring normal physiological function. However, unlike *Hypnea*, ryegrass (*Lolium*) responds differently to Pb stress; when exposed to 500 μM Pb, the carotene, chlorophyll a, and chlorophyll b levels in the plants, as well as the photosynthesis rates were significantly lower than in the control plants. Among the parameters studied, the net photosynthesis (Pn) and transpiration rates (Tr) were decreased by 52.9 and 39.4%, respectively [17]. Similarly, under conditions of Pb stress, the growth of *Robinia pseudoacacia* (black locust) seedlings was repressed, and Pb ions were found to be mostly concentrated in the roots [18]. At a Pb concentration of 2000 mg kg^-1^ in soil, the Pb concentration in the root was approximately 400 μg g^-1^ [13], and the number of chloroplasts and photosynthesis ability decreased significantly; at Pb concentration of 1400 mg kg^-1^ of the soil, the seedling Pn, Fv/Fm, and quantum efficiency of PSII (ΦPSII) were decreased by 60.62, 29.2, and 55.07%, respectively [11]. However, the addition of *Funneliformis mosseae* and *Rhizophagus intraradices* to black locust seedlings under conditions of Pb stress could facilitate photosynthesis as well as their ability to remove reactive oxygen species, and decrease the level of Pb ions in the leaves; it also significantly attenuated the toxic effects of Pb ions [13]. Under conditions of Pb stress, the photosynthetic ability of plants is weakened because of physiological damage, thereby, threatening their survival.

*Ligustrum lucidum* Ait. (privet) is a species of privet belonging to the family Oleaceae, which originated in the Yangtze River basin in China. The northern extent of its natural dispersion range extends northward from the Huai River to the Qin Mountains. Big-leafed privet plants have the added advantages of being an evergreen tree and being tolerant to drought [19], low temperatures [20], smoky conditions, dust, and environmental pollution [21], and are commonly planted as sidewalk and landscaping trees. In Palermo city, with a Mediterranean climate, privet plants grow very well, but the Pb concentration in leaves sampled from different sites, was found to fluctuate almost from 2.5 to 7.5 mg kg^-1^, which was significantly different between sites with different traffic levels [22]. Therefore, privet plants, in this Mediterranean city, were proposed as a bioindicator for air pollution derived from traffic emission. Similarly, in Changchun city of China, Pb concentration in privet leaves was observed to range from 11.4 to 20.0 mg kg^-1^ at different sites, and high Pb content in leaves was found to be associated with high level of traffic [23]. Privet plants show strong tolerance to Pb pollution and can be used for removing Pb pollution in the urban environment caused by traffic. In addition, privet plants are used in bioremediation, especially of soil pollution caused by mining, as they demonstrate a stronger ability for accumulation and transport of Pb ions. Kang et al. [24] reported that the concentration of Pb ions in the Tonglushan ancient copper mine in the Hubei province of China was 137.06 mg kg^-1^; the concentration of Pb ions in the roots of naturally growing privet plants in the region was 61.8 mg kg^-1^, whereas the concentration in the soil was 33.0 mg kg^-1^, indicating that its enrichment and transport coefficients were 0.45 and 0.53, respectively. In the Pb and zinc mines in Zixing, Hunan Province, the enrichment and transport coefficients of the naturally growing privet plants were as high as 0.107 and 1.09, respectively [25]. In the Pb and zinc mines of the Fuyang district, Zhejiang Province, Chen et al. [26] planted a number of species, including privets, to restore the polluted soil, and reported that the enrichment concentration of Pb ions in the privet roots was approximately 300 mg kg^-1^, whereas its ion transfer coefficient was 0.65. The levels of Pb in the polluted soil decreased significantly through bioremediation. Li et al. [27] reported that after 3 years of privet cultivation, the removal rate of Pb from the lead and zinc ore was 27.31%. The enrichment and transport effects of privet on the Pb pollutants might be attributed to the concentration of Pb in the soil. At present, the research on the use of privet plants for soil restoration is in preliminary stages, and mostly focuses on the enrichment of Pb in naturally occurring plants as well as on long-term surveys, and does not involve the photosynthetic and cellular mechanisms underlying the observed tolerance of privet trees.

This study was aimed at accurately measuring the seedling growth, enrichment of Pb ion, photosynthesis, and other physiological characteristics in privet plants, and seeking evidence for these characteristics on an ultrastructural cellular level. Moreover, we performed multiple linear regression analysis to confirm the causes and the extent of the effect of Pb on the development and photosynthetic mechanisms of seedlings, so as to determine a basis for future application of privet seedlings in the restoration of soils polluted with heavy metals.

## Materials and methods

### Plant material and treatments

Five concentrations (0, 200, 600, 1000, and 1400 mg kg^-1^ soil) of Pb, supplied as lead nitrate mixtures, were used to treat the soil (pH = 8.29 ± 0.03). The seeds of privet were soaked in warm water at 50°C for 48 h, and then buried in 60% damp sand to germinate for seven days. Turgid seeds were selected, and sown on the prepared soil; five seeds were planted in each pot containing 3.5 Kg of soil, and were covered with about 1 cm of soil; six pots were prepared for each of the Pb treatments. After germination, Hoagland solution at ¼ concentration (pH = 5.46 ± 0.13) was used for watering, every two weeks; the seedlings were placed in a greenhouse, with temperature settings of 28°C during the day, and 25°C during the night. During the seedling stage, the seedlings were monitored and the weak plants were discarded; it was ensured that only one to two seedlings remained in each pot. When the seedlings were 10-months-old, those exposed to low and middle concentration of Pb grew normally, whereas those exposed to high concentration showed growth inhibition to a certain extent (S1 Fig). Some representative seedlings, for example, long, medium, and short, were selected from each treatment, and were subjected to the relevant measurements of the growth indicators and tissue sectioning.

### Measurement of growth indicators

A measuring tape and calipers were used to measure the seedling height and stem diameter. An electronic balance (with sensitivity in milligrams) was then used to measure the dry and fresh weights of the seedlings. The seedlings were placed in a container, and transferred to the oven after taking their fresh weights. The seedlings were fixed at 105°C for 5 min, and placed at a constant temperature of 70°C for 12 h, until they were dried to a constant weight. The plants were then weighed, and the process was repeated four times.

### Measurement of the concentration of Pb ions

The dried plants were retrieved, crushed, and ground, and the powder was placed in a beaker. About 7 mL of nitric acid and 2 mL of hydrogen peroxide were added to the beaker, which was then placed in a microwave oven at 170°C for 30 min. The homogenate was then retrieved, and transferred to a beaker with polytetrachloroacetic acid containing 20% dilute nitric acid by volume. To remove the acid content, the mixture was heated at 170°C on an electric heating pad until it was almost dry. The residue was then transferred to a 25 mL bottle and the volume was adjusted using 20% dilute nitric acid by weight. Finally, an Optima 2100 DV inductively coupled plasma mass spectrometer (PerkinElmer, USA) was used to conduct a full-scale analysis on Pb ions.

### Extraction of plastid pigments and determination of their concentrations

Pigments were extracted from the plastids using mixed liquid extraction [28]. About 0.2–0.5 g portions were weighed and cut into smaller pieces, and the process was repeated four times. Acetone and ethanol were mixed at 2:1 ratio to produce 12 mL of a solution, into which the fragments were transferred. The mixture was placed under dark conditions for 12 h, until the structures turned white. The colorimetric analysis was performed on the extracted liquid using a 722 spectrophotometer (Shanghai Jingke Electronic Co., Ltd., China); the wavelengths used were 664, 662, 644, and 440 nm, respectively, and the pigment concentrations were calculated as follows [29]:

$$C_{a}=9.78\times OD662-0.99\times OD644$$

$$C_{b}=21.43\times OD664-4.56\times OD662$$

$$C_{a+}{}_{b}=5.13\times OD662+20.44\times OD664$$

$$C_{k}=4.7\times OD440+0.27\times C_{a+b}$$

where C_a_ is the chlorophyll a concentration, C_b_ is the chlorophyll b concentration, C_a+b_ is the total chlorophyll concentration, C_k_ is the carotenoid concentration, and OD is the absorbance at the specified wavelength.

### Measurement of the photosynthetic parameters

The net photosynthesis (Pn), transpiration rate (Tr), stomatal conductance (Gs), and sub-stomatal CO_2_ concentration (Ci) in the privet seedlings exposed to the different treatments were measured using an Li-6400 portable photosynthesis system (Li-Cor, USA). An internal light source was used to supply the light energy, and CO_2_ was pumped into the system to maintain its constant level. The internal light source was set at an intensity of 1000 μmol m^-2^s^-1^, and the CO_2_ concentration was maintained at 400 μmol mol^-1^. In the measurement process, the intact and fully- expanded leaves were selected for determination from upper part of the seedlings. All the parameters were determined using five repetitions.

### Measurement of chlorophyll fluorescence

We used a Beijing Yaxin-1161G chlorophyll fluorescence analyzer/fluorescence meter (Beijing Yaxin Liyi Technology Co., Ltd, China) to measure the minimum fluorescence (Fo), maximum photochemical efficiency (Fv/Fm), photochemical quenching (qP), non-photochemical quenching (qN), and quantum efficiency of photosystem II (ΦPSII). The leaves were placed in darkness for 30 minutes prior to the measurements, after which the saturation pulse method was applied—saturated pulses of light (3000 μmol m^−2^ s^−1^) were applied for 1 s, followed by the light from a modulated light source (1500 μmol m^−2^ s^−1^) for 9 s. Finally, a light-induced curve was used to measure the chlorophyll fluorescence. For the measurement, undamaged leaves were selected from the mid- to the top-portions of the seedlings. All the parameters were determined using five repetitions.

### Cell ultra-slice treatment of leaves and structural observations

Five leaves were taken and quickly placed in 0.4% glutaraldehyde solution. Subsequently, 1 mm wide and 2 mm long sections were taken following the lateral veins, throughout the entire lateral vein. The sections were fixed in 0.4% glutaraldehyde for 20 h, and then 0.1 mol L^-1^ phosphoric acid was used to wash the samples. At 6°C, 1% osmium acid was used to fix the samples for 6 h; thereafter, 0.1 mol L^-1^ phosphoric acid was used to wash the samples. Acetone was used for dehydration; the dehydrated samples were soaked in a mixture of acetone and Epon812 (1:1, 1:2, v/v, 30 min), and were finally embedded in Epon812. The embedded samples were polymerized in 30°C and 40°C ovens for one day each, and finally under a 60°C environment for 3 days. The embedded samples were trimmed, and an LKB-V ultra-thin slicer (LKB Instruments, Sweden) was used to make thin slices of the samples. Uranium acetate and citric acid lead were used to double stain the samples. Finally, a HITACHI H-600 transmission electron microscope (Hitachi High-Technologies, Japan) was used to observe the cellular changes, and record the videos.

### Statistical analysis

We used SPSS 21.0 to perform variance analysis and Duncan multiple comparison, multiple linear regression analysis, and linear correlation analysis. A value of *P* < 0.05 indicates statistical significance in the multiple comparison, and denotes significant correlation. A value of *P* > 0.05 denotes an absence of significance in the multiple comparison, and no significant correlation.

## Results

### Growth characteristics of privet seedlings

**Fig 1:**
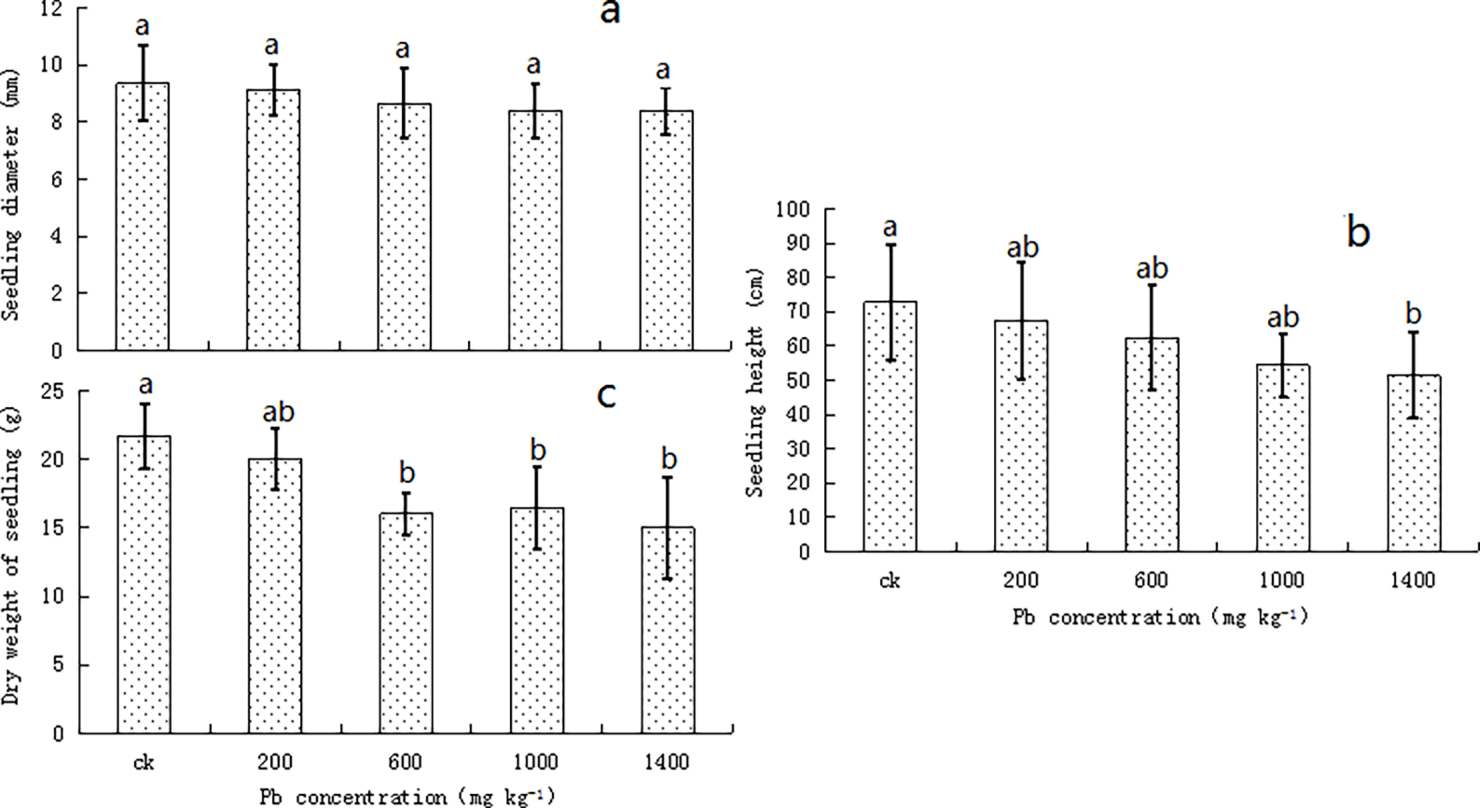
Effect of Pb concentrations in the soil on the growth characteristics of *Ligustrum lucidum* seedlings. (a) stem diameter, (b) seedling height, and (c) dry weight. Vertical bars indicate means ± SD, n = 4. ANOVA values with different letters are significantly different (*P* < 0.05).

With the increase in Pb concentration, the ground diameter and height of the privet seedlings gradually decreased, but the differences were not significant between the consecutive treatments (*P* > 0.05; see Fig 1A and 1B). At a concentration of 1400 mg kg^-1^, the ground diameter and height of the plants were the lowest, and they were decreased by 10.39% and 29.31%, respectively, compared to the corresponding values in the control. As the Pb concentrations increased, the dry weight of the plants gradually decreased (Fig 1C), being significantly lower (*P* < 0.05) by 26.16, 24.18, and 30.78%, respectively, than the corresponding values in the control plants at 600, 1000, and 1400 mg kg^-1^ concentrations.

### Lead concentrations in the privet seedlings

**Fig 2:**
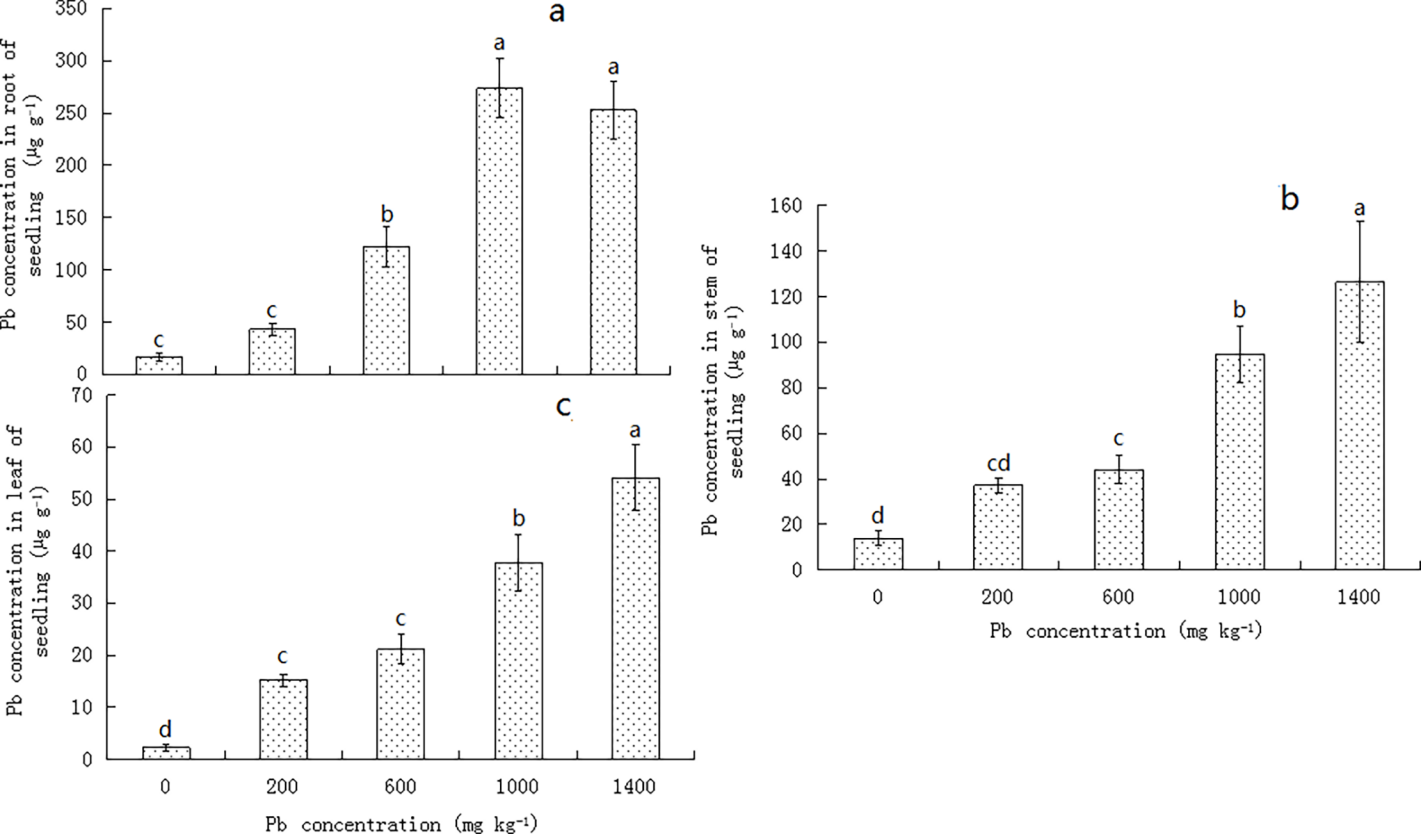
Effect of lead concentrations in the soil on the lead concentrations in *Ligustrum lucidum* seedlings. (a) root, (b) stem, (c) leaf. Vertical bars indicate means ± SD, n = 3. ANOVA values with different letters are significantly different (*P* < 0.05).

The Pb concentrations in the roots, stems, and leaves of privet seedlings showed an increasing trend following an increase in Pb concentration in the soil, with only the concentration in the roots being slightly lower in the presence of 1400 mg kg^-1^ Pb than in the presence of 1000 mg kg^-1^ Pb; however, this was not statistically significant (*P* > 0.05). At intermediate and high Pb concentrations (≥ 600 mg kg^-1^), the concentrations in both the privet roots and stems were higher than in the control levels (*P* < 0.05), with the highest concentrations present in the 1000 and 1400 mg kg^-1^ treatments, which were 15.54 and 8.07 fold of their values in the control, respectively (Fig 2A and 2B). In the leaves, all the treatments yielded higher concentrations of Pb compared to that in the control, with the highest Pb concentrations reaching 54.05 μg g^-1^ in the 1400 mg kg^-1^ treatment (Fig 2C).

### Pigment content in the privet seedlings

**Fig 3:**
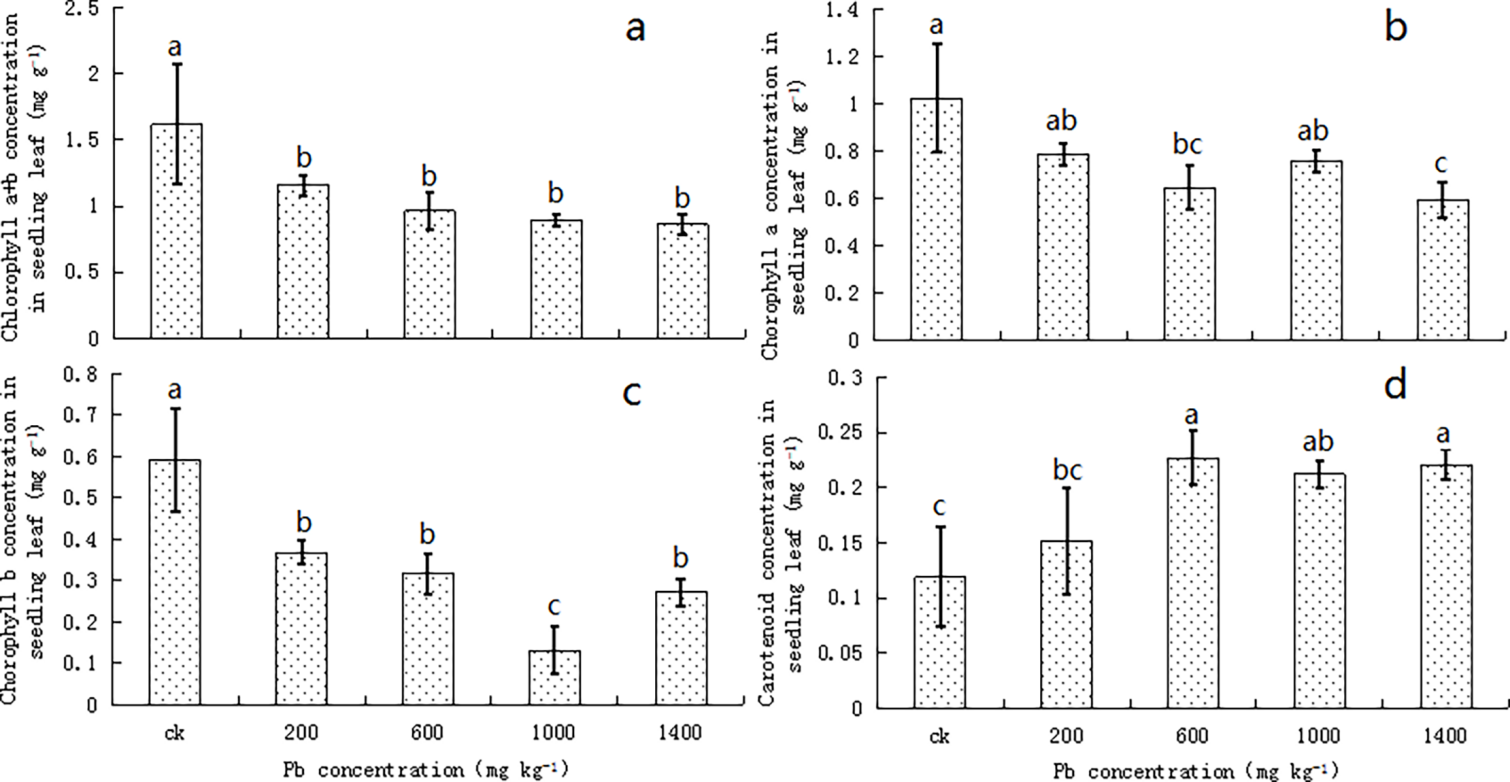
**Variations in the Contents of Total Chlorophyll (a), Chlorophyll a (b), Chlorophyll b (c), and Carotenoids (d) in *Ligustrum lucidum* Seedlings under Lead Stress.** Vertical bars in the figure indicate means ± SD, n = 3. Different letters indicate a significant difference at *P* < 0.05.

The content of total chlorophyll, chlorophyll a, and chlorophyll b showed similar fluctuations; with the increase in Pb concentrations, the values for all the three showed decreasing trends. Under the different conditions of Pb stress, the total chlorophyll and chlorophyll b contents were significantly lower than the control values (*P* < 0.05; Fig 3A and 3C); however, the chlorophyll a levels were lower in the seedlings under Pb stress than in the control, but showed no statistical significance (*P* > 0.05; Fig 3B) and the differences were 23.24% and 26.17%, respectively, in the 200 and 1000 mg kg^-1^ treatments compared to the control value.

Under lead stress, the carotene levels in the privet seedlings increased with the increase in lead concentration, showing an opposite trend compared to that observed for the chlorophyll levels (Fig 3D). At moderate to high Pb concentrations (≥ 600 mg kg^-1^), the carotene levels in the seedlings were significantly higher than in the control (*P* < 0.05); it increased to 0.22 mg g^-1^, which was 85.71% higher than the control value, in the 1400 mg kg^-1^ treatment.

### Photosynthetic ability of the privet seedlings

**Fig 4:**
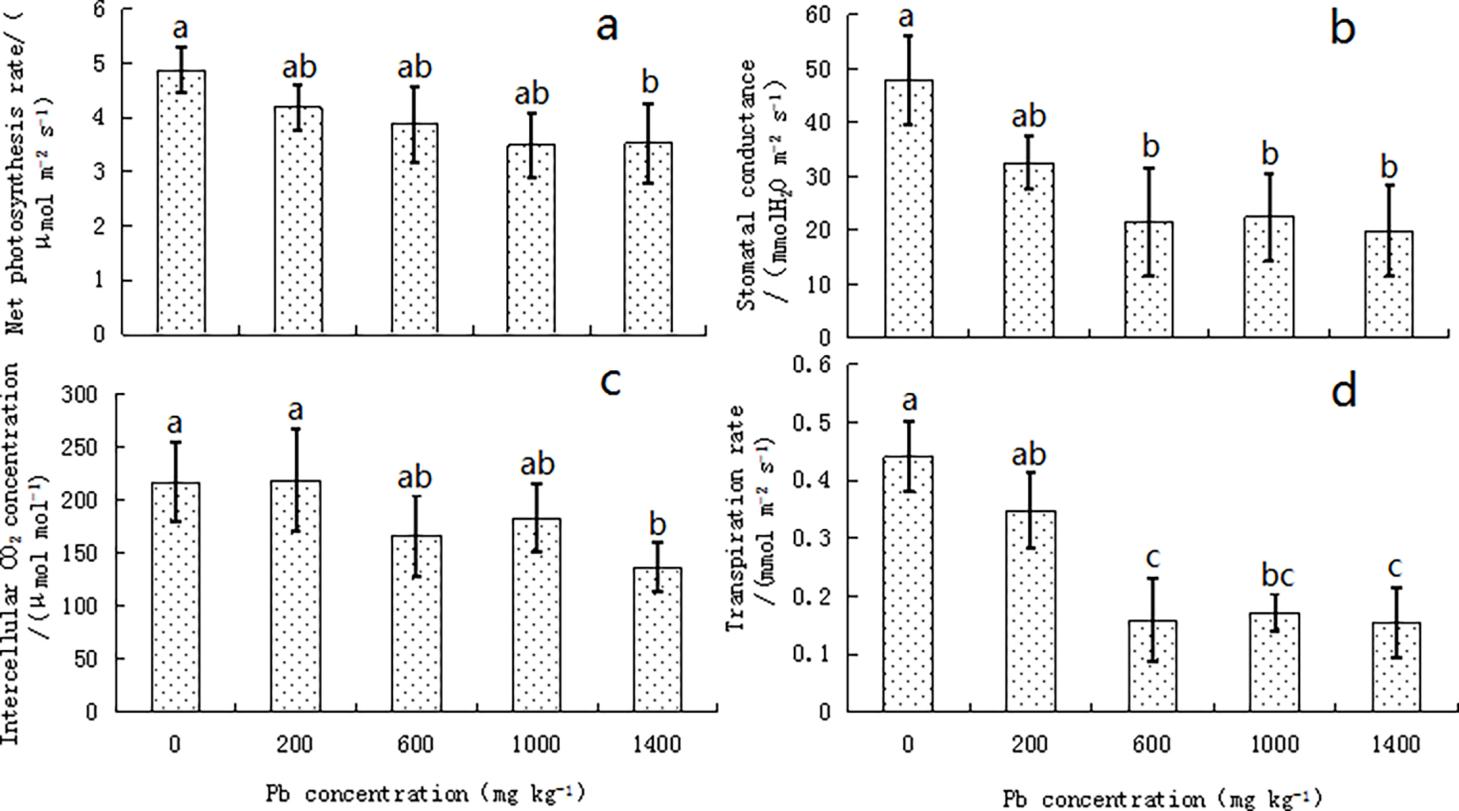
**Variation in the Net Photosynthetic Rate (Pn; a), Stomatal Conductance (gs; b), Intracellular CO**_**2**_ **Concentration (Ci; c), and Transpiration Rate (Tr; d) of *Ligustrum lucidum* Seedlings under Lead Stress.** Vertical bars in the figure indicate means ± SD, n = 5. Different letters indicate significant differences at *P* < 0.05.

The values of Pn, Gs, Tr, and Ci showed similar changes, and largely decreased with the increased Pb concentrations (Fig 4). The values of Pn and Ci were lower in the seedlings under the lead stress compared to that in the control; those in the 1400 mg kg^-1^ treatment were 3.52 μmol m^-2^ s^-1^ and 137.36 μmol mol^-1^, respectively, and differed significantly from the control values; however, the differences in the other treatments were not significant (*P* > 0.05; Fig 4A and 4C). At moderate to high Pb concentrations (≥ 600 mg kg^-1^), Gs and Tr of the seedlings were significantly lower than the control values (*P* < 0.05), but did not differ significantly with each other (*P* > 0.05; Fig 4B and 4D); their lowest values were 19.95 mmol H_2_O m^-2^ s^-1^ and 0.15 mmol m^-2^ s^-1^, respectively, in the 1400 mg kg^-1^ Pb treatment.

### Chlorophyll fluorescence of the privet seedlings

**Fig 5:**
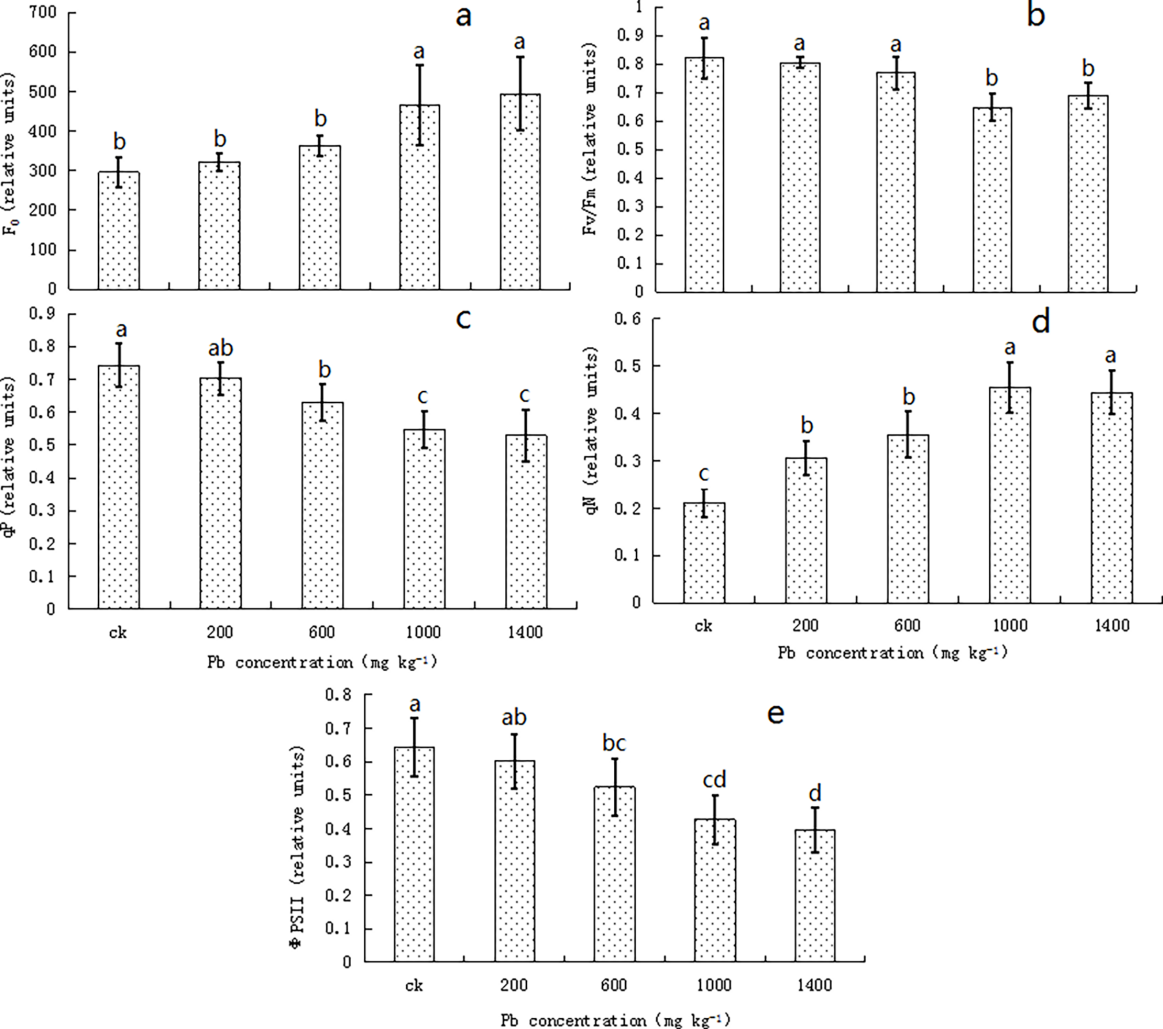
**Effects of Lead Stress on the Initial fluorescence (F**_**0**_**; a), Maximum Photochemical Efficiency (F**_**v**_**/F**_**m**_**; b), Photochemical Quenching (qP; c), Nonphotochemical Quenching (qN; d), and Quantum Yield (ΦPSII; e) of *Ligustrum lucidum* Seedlings.** Vertical bars in the figure indicate means ± SD, n = 5. Different letters indicate significant differences at *P* < 0.05.

With the increase in Pb concentrations, the minimum fluorescence (F_0_) and non-photochemical quenching (qN) showed increasing trends (Fig 5A and 5D), but in the 200 and 600 mg kg^-1^ treatments, F_0_ was not significantly different between the treatment and control groups (*P* > 0.05). The peak values for F_0_ and qN were obtained in the 1400 and 1000 mg kg^-1^ treatments; the values were 439.80 and 0.454, respectively, and were higher by 66.75% and 113.43%, respectively, compared to that in the control seedlings.

In contrast to the above observations, the maximal photochemical efficiency (Fv/Fm) showed a decreasing trend (Fig 5B), with the seedlings in the 1000 and 1400 mg kg^-1^ treatments showing a significantly lower value of Fv/Fm than in the control (*P* < 0.05); the lowest value was observed in the 1000 mg kg^-1^ treatment; it was 0.648 and was lower by 15.85% than the control value.

The values of qP, ΦPSII, and other such factors decreased following an increase in the Pb stress (Fig 5C and 5E). At moderate to high lead concentrations (≥ 600 mg kg^-1^), the values of qP and ΦPSII in the stressed seedlings were significantly lower than in the control (*P* < 0.05); the lowest values were observed in the 1400 mg kg^-1^ treatment, which were lower by 28.86 and 38.46%, respectively, than their control values.

### Correlation between the biomass growth, photosynthetic ability, and other factors in the privet seedlings

Linear correlation analysis showed that the growth of Pb-stressed plants measured in terms of the dry weight (DW) correlated extremely significantly with the Pb concentrations in the roots (Pb_root_), stems (Pb_stem_), and leaves (Pb_leaf_), and with the transport coefficients of Pb ions (stems/roots) (Pb_stem/root_) (*P* < 0.01). It was significantly correlated with the transport coefficients of lead ions (leaves/stems) (Pb_leaf/stem_) (*P* < 0.05; see Table 1). The results showed that the biomass accumulation in the privet seedlings under Pb stress was affected by the level of Pb ions in the tissue and by the transport of Pb ions. In addition, the values of Pn, Gs, Tr, Ci, Fv/Fm, qP, and ΦPSII were significantly correlated (*P* < 0.01; Table 1), showing that the Pn of seedlings under Pb stress was not only affected by the external conditions, but also by the systemic photosynthesis ability.

**Table 1:** Linear correlations between the dry weight (DW), photosynthetic function (Pn), and their influencing factors in *Ligustrum lucidum* seedlings under lead stress.

Factor	Pb_root_		Pb_stem_			Pb_leaf_		Pb_stem/root_			Pb_leaf/stem_	
Pearson Correlation Coefficient with DW	-0.620**		-0.542**			-0.615**		0.704**			-0.427*	
Factor	Gs	Tr		Ci	F_0_		Fv/Fm		qP	qN		ΦPSII
Pearson Correlation Coefficient with Pn	0.665**	0.678**		0.529**	-0.057		0.654**		0.821**	-0.213		0.832**

* and ** show significant correlation (*P* = 0.05) and extremely significant correlation (*P* = 0.01), respectively.

By comparing DW with the five indicators, namely Pb_root_, Pb_stem_, Pb_leaf_, Pb_stem/root_, and Pb_leaf/stem_, Pn with the three indicators, namely Gs, Ci, and Tr, and the five indicators, F_0_, Fv/Fm, qP, qN, and ΦPSII, and performing a multiple linear regression analysis, we obtained three distinct multiple linear regression models, as follows:

$$y_{1}=5.070-0.103x_{1}+1.308x_{2}-1.826x_{3}+1.159x_{4}-0.147x_{5};\;R^{2}=0.649$$

$$y_{2}=3.612+0.600x_{1}+0.609x_{2}+0.727x_{3};\;R^{2}=0.477$$

$$y_{3}=-0.113+0.799x_{1}-1.738x_{2}+1.182x_{3}+0.370x_{4}+1.710x_{5};\;R^{2}=0.815$$

Here, *y*_1_ is DW, and x_1_, x_2_, x_3_, x_4_, and x_5_ are Pb_root_, Pb_stem_, Pb_leaf_, Pb_stem/root_, and Pb_leaf/stem_, respectively; *y*_2_ is Pn, and x_1_, x_2_, and x_3_ are g_s_, Ci, and Tr, respectively; *y*_3_ is Pn, and x_1_, x_2_, x_3_, x_4_, and x_5_ are F_0_, Fv/Fm, qP, qN, and ΦPSII, respectively.

In the multiple linear regression analysis, the absolute values of the standardized regression coefficients (SRC) reflect their respective effects on the comprehensive index [11]. Based on regression Model 1, the SRC value of Pb_leaf_ was the highest at 2.153; the SRC value of Pb_stem_ was second highest at 1.845 and that of Pb_stem/root_ was the third highest at 0.378. The SRC values of Pb_leaf/stem_ and Pb_root_ were similar, they being the lowest (Fig 2). In Model 2, Tr had the highest SRC value of 0.342, that of g_s_ was the second highest, and the SRC value of Ci was the third highest (Table 2). In Model 3, qP and ΦPSII had the SRC values of 0.745 and 0.617, ranking first and second, respectively; the SRC values of Fv/Fm had an absolute value of 0.424, ranking third, which was followed by F_0_; the SRC value of qN was the smallest, and ranked fifth (Table 2).

**Table 2:** Multiple linear regression analyses of biomass accumulation and photosynthetic functions in *Ligustrum lucidum* seedlings under lead stress conditions, considering the factors that influence the photosynthesis and chlorophyll fluorescence indices.

Regression model	*F*-value	*P* value	Standardized regression coefficients				
			*b*_1_	*b*_2_	*b*_3_	*b*_4_	*b*_5_
Model 1	5.174	0.007	-0.073	1.845	2.153	0.378	-0.029
Model 2	6.395	0.003	0.264	0.131	0.342		
Model 3	16.754	0.000	0.188	-0.424	0.745	0.099	0.617

b_1_–b_5_ are the standardized regression coefficients of x_1_–x_5_ from the multiple linear regression equations

### Ultrastructural characteristics of the privet seedling leaves

**Fig 6:**
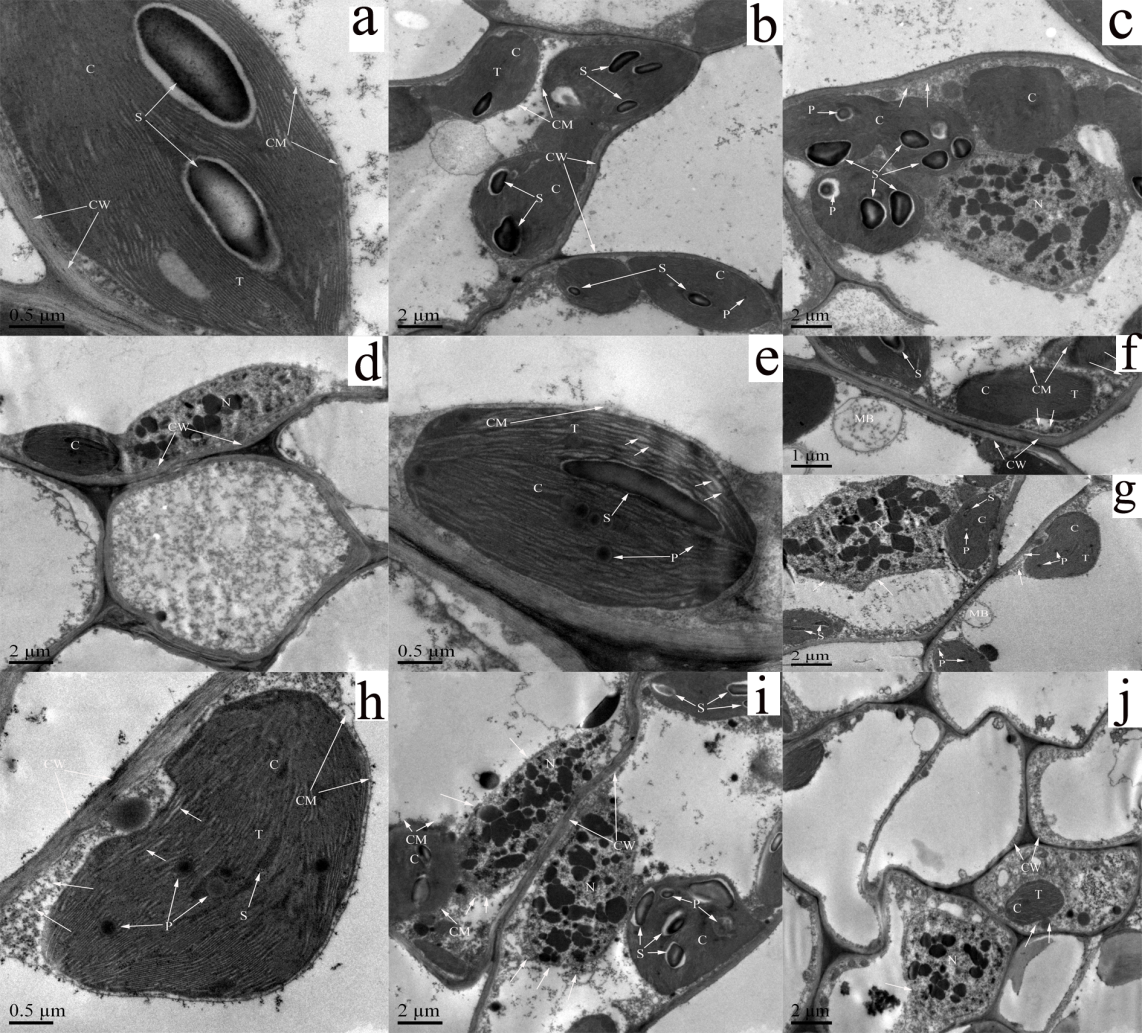
Ultrastructure of cells in the leaves of *Ligustrum lucidum* seedlings under different lead concentrations. (a) 0 mg kg^-1^, (b, c) 200 mg kg^-1^, (d, e, f) 600 mg kg^-1^, (g, h) 1000 mg kg^-1^, and (I, j) 1400 mg kg^-1^. C, chloroplasts; CM, chloroplast envelope; CW, cell wall; N, nucleus; MB, multivesicular body; P, lipid globules; S, starch grain; T, thylakoid lamellae. Scale bars: 0.5 μm in a, e, and h; 1 μm in f; 2 μm in b, c, d, j, h, and i.

In the control and 200 mg kg^-1^ treatment, the chloroplasts and their sub-organelles were normal. The chloroplasts were in close proximity to the cell walls, and their outer membranes were clear. They contained starch grains and small lipid globules; the thylakoid lamellae were neatly arranged in rows and were clearly observed (Fig 6A, 6B and 6C). However, under medium and high-level stress, the shapes of chloroplasts and their sub-organelles changed gradually. The chloroplasts with slight plasmolysis (indicated in the figure by arrows) and swelling contained starch grains and lipid globules, their outer membranes were indistinct, and thylakoid lamellae were arranged slight haphazardly and appeared to be expanding (see the arrows in Fig 6E) in the 600 mg kg^-1^ treatment. When lead concentration increased to 1000 mg kg^-1^, the chloroplasts were swollen and still contained starch grains and lipid globules; some cell wall separation was observed (pointed by arrows in Fig 6H). Parts of the outer membrane were missing, and furthermore, the thylakoid lamellae were arranged haphazardly and were expanding (pointed by arrows in Fig 6H). However, the chloroplasts were severely swollen, and some were even completely separated from the cell wall (indicated by arrows in Fig 6J), but still contained starch grains and lipid globules, and the outer membranes were apparently missing in the 1400 mg kg^-1^ treatment.

In this experiment, the cell wall distribution appeared normal in the control and 200 mg kg^-1^ treatment (Fig 6A, 6B and 6C). With an increase in lead concentration, the cell walls stabilized and were generally normal under medium and high Pb concentrations (Fig 6D, 6I and 6J). In contrast, the nucleus showed obviously variations under lead stress. Under low and medium lead concentrations, the nucleus showed some signs of shrinkage (Fig 6B, 6C and 6D). When Pb concentration increased to 1000 mg kg^-1^, the nucleus showed shrinkage and some deformation (Fig 6G). Furthermore, the cell nucleus were further shrunken in the 1400 mg kg^-1^ treatment (Fig 6I). In addition, the mesophyll cells appeared to contain a multivesicular body in the 600 and 1000 mg kg^-1^ treatment (Fig 6F and 6G), whereas, this temporary organelle was absent in the other treatments during this experiment.

## Discussion

### Growth and resistance of privet seedlings to lead stress

We observed that the growth of privet seedlings was suppressed in the different treatments; in the 1400 mg kg^-1^ treatment, the ground diameter, height, and dry weight of the stressed seedlings were 89.6, 70.7, and 69.3% of their corresponding values in the control plants, showing that the privet seedlings were resistant to Pb stress. Many tree species, including the salt tree, are used in the bioremediation of polluted soils because of their resistant properties [30]. Shi et al. [31] reported that in the presence of 1000 mg kg^-1^ Pb, the Pb concentration in the top portions of salt trees was 13.50 μg g^-1^, and that in the roots was 25.00 μg g^-1^. Under the same conditions, the Pb concentrations in the roots and stems were 273.60 and 94.5 μg g^-1^, respectively. Therefore, privet seedlings have good potential for bioremediation, and can be used directly to treat Pb-polluted soils under suitable conditions.

Lead enrichment in privet seedlings is in the order, roots > stems > leaves, which is similar to that in plants like *Armeria maritima*, *Agrostis tenuis*, and *Cardaminopsis halleri* [32]. The ion enrichment occurs in plant roots, and they are then transferred to stems and leaves. The biological toxicity of Pb ions blocks cellular mitosis, affecting the growth of roots and stems [14]; in leaves, it disrupts photosynthesis in a number of ways, such as by causing structural deformities, suppressing the formation of chlorophyll, blocking the electron transport, and closing the stomata to reduce the CO_2_ usage efficiency [33], and, thereby, adversely affects the plant development. In this study, based on the SRC values obtained from MRA Model 1, the Pb ions were found to disrupt photosynthesis in leaves; the blockage of organic synthesis had the greatest effect on the development of privet, in line with our test results. In stems, the Pb ions disrupt mitosis, causing secondary disruption of privet growth, whereas in roots, these ions have the least effect on the seedling growth, possibly because of their cellular structure. For instance, the cell walls of the root cells can stabilize the Pb ions [34], large amounts of these ions can accumulate in the intercellular spaces in the form of insoluble oxalates, phosphates, and chlorates [35], and the aqueous environment can act as an insulation against active lead [36], effectively decreasing the biological toxicity of the Pb ions. With regard to the transport of Pb ions, it was higher between the stems and roots of the privet seedlings compared to that between the leaf and stem, probably because of the respective transport modes. Besides using the symplast pathway, Pb ions can also enter the inner cellular membranes in the roots and accumulate there through the apoplast pathway; in this case, the casparian strip gates the upward transport of lead ions toward higher tissues [37], allowing for better control of plant development.

### Photosynthetic ability of privet seedlings under lead stress

Under stressful environmental conditions, both stomatal and non-stomatal factors can decrease the photosynthetic abilities of plants [38]. In this study, based on the SRC values obtained from MRA Model 2, the transpiration rate (Tr) was observed to have the maximum effect on the photosynthetic ability, possibly as a result of the upward transport of Pb ions through evaporation [39]. Apart from the fixation of Pb ions in the surrounding tissues of the vessels, the excess ions accumulate in the cuticular layer of leaves [40], thereby, affecting the photosynthesis. The secondary effect on photosynthesis is caused by the stomatal conductance (gs). The stomatal factors are the second largest factors affecting photosynthesis; sub-stomatal CO_2_ concentration (Ci) has the least effect on photosynthesis, presumably because of CO_2_ absorption, and is significantly correlated with the CO_2_ assimilation efficiency [41], providing carbon for photosynthesis [42].

In terms of chlorophyll fluorescence, when the reaction center of photosystem II (PSII RC) opens, the primary electron acceptor of PSII (Q_A_) is completely oxidized, and the fluorescence emitted at this time is F_0_. In this study, we observed that an increase in F_0_ reflected the gradual inactivation of PSII RCs [43]. When PSII RC closes, Q_A_ is completely reduced, and the fluorescence at this time is F_m_; F_v_ denotes the difference between F_m_ and F_0_. We observed that the value of F_v_/F_m_ gradually decreased, reflecting the gradual inactivation of PSII RCs, dissipation of a large amount of excitation energy (EE) in the form of heat [43, 44], or a blockage of electron transport [45]. In addition, qP indicates the excited energy used in photosynthesis, and also reflects the stress induced by excess EE on PSII [46]; excess activation energy is a major cause of PSII destruction [47]. qN denotes the amount of excess energy dissipated as heat, through a process which depends on lutein [16], to avoid PSII damage caused by the excess activation energy [46]. In the present study, qP decreased in the Pb-stressed seedlings, whereas qN increased, indicating that the activation energy involved in photosynthesis was decreased, pressure from the excess activation energy was increased, and the activation energy that was dissipated as heat was also consequently increased. This result is in line with the observed changes in carotene aimed at protecting the PSII and ensuring the stability of photosynthetic function. ΦPSII is closely linked to the electron transport efficiency of the non-cyclic electron transport chain in PSII [48], reflecting the state of the PSII electron chain [47]. In this experiment, ΦPSII gradually decreased in the privet seedlings under Pb stress, showing that the electron transport was restricted. Based on MRA Model 3, it can be concluded that the activation energy is involved in the decrease in photosynthesis, and gradually exerts a greater impact on the photosynthetic ability of the privet plants. The blockage of the electron transport is the second most influential factor and a decrease in the PSII RC activity is the third most important factor, whereas the dissipation of excess activation energy through heat has the least effect on photosynthesis.

### Ultrastructural characteristics of privet plants under lead stress

In this study, significant changes were observed in the ultrastructural characteristics of chloroplasts. The chloroplasts were swollen and demonstrated gradual loss of their outer membrane. The inner membrane was expanded and separated from the cell wall, resulting in abnormalities in the structure, and an overall decrease in the photosynthetic ability. Based on these observations, we can conclude that the cellular structure reflects the extent of damage caused by Pb in the privet seedlings. In addition, we observed multivesicular bodies in the seedling cells only in the 600 mg kg^-1^ treatment. These multivesicular bodies probably originate from the Golgi bodies and can transport or provide cell-building molecules, such as hemicellulose and gelatin [49]. Xu et al. [50] investigated 58,000 proteins in *Arabidopsis thaliana*, and reported that these multivesicular bodies were involved in the construction of cell wall structures. In this study, these multivesicular bodies were found to be affected by the Pb stress; their absence in the privet cells under other treatments could be attributable to the biological toxicity conferred by the Pb stress. The formation of multivesicular bodies aids the cell wall repair and protection functions, and ensures the complete preservation of the structure and function of cell walls, and might be a special adaptation of privet seedlings to lead-polluted soil.

## Conclusions

In this study, Pb pollution was observed to inhibit the growth capacity of privet seedlings, weakened their photosynthetic function as evident from the photosynthesis indices and chlorophyll fluorescence indices, and decreased their chlorophyll content. In addition, the structure of cellular organelles was changed, with chloroplasts showing the most drastic changes, like plasmolysis and swelling of thylakoid lamellae. The lead content of the leaves was the main factor affecting the growth capacity; however, the activation energy in photosynthesis, the damage caused by excess activation energy, and transpiration rate had the maximum effect on the photosynthetic function in the lead-stressed privet seedlings. Our results suggest that privet has strong potential for use in bioremediation, and could be widely used for Pb-polluted soils under suitable conditions.

## Supporting information

S1 Fig The growth state of 10-month-old privet seedlings placed together in greenhouse under lead stress. (TIF)
