# Supplementary figures and images for: Effects of lead stress on the growth, physiology, and cellular structure of privet seedlings
Source: PLoS One. 2018 Mar 1;13(3):e0191139. doi: 10.1371/journal.pone.0191139 (PMC5832220; doi:10.1371/journal.pone.0191139)

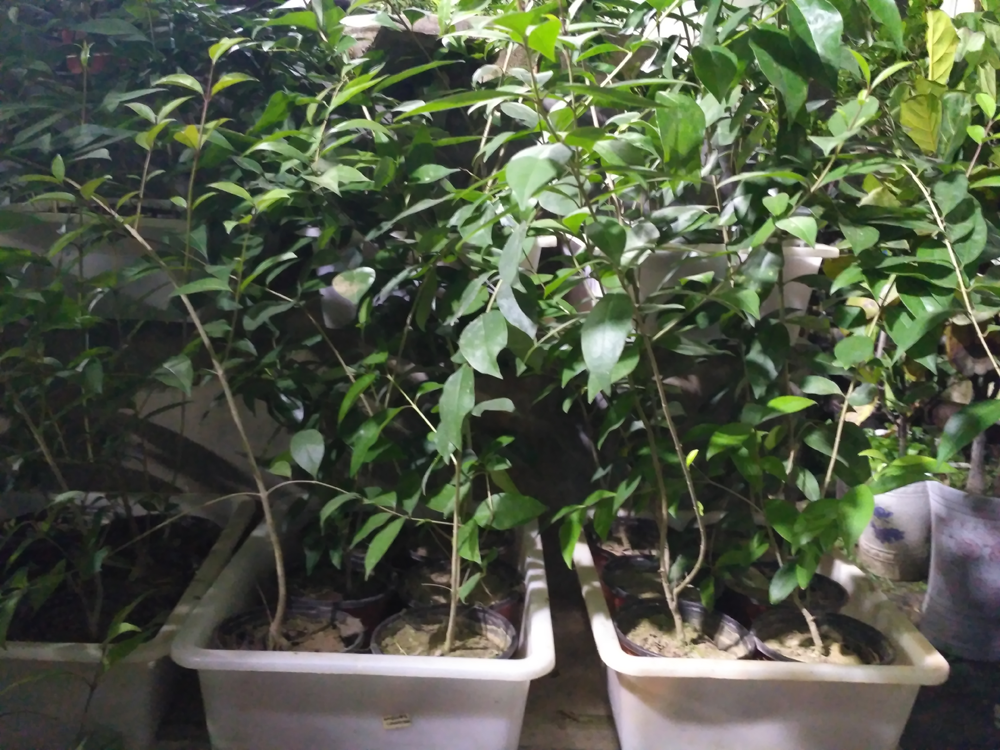

Supplement: S1 Fig — (TIF) [file pone.0191139.s001.tif]
